# Discovery of a Lipid Synthesising Organ in the Auditory System of an Insect

**DOI:** 10.1371/journal.pone.0051486

**Published:** 2012-12-12

**Authors:** Kathryn F. Lomas, David R. Greenwood, James FC. Windmill, Joseph C. Jackson, Jeremy Corfield, Stuart Parsons

**Affiliations:** 1 School of Biological Sciences, University of Auckland, Auckland, New Zealand; 2 The New Zealand Institute for Plant & Food Research Limited, Auckland, New Zealand; 3 Centre for Ultrasonic Engineering, Department of Electronic and Electrical Engineering, University of Strathclyde, Glasgow, United Kingdom; CNRS, France

## Abstract

Weta possess typical Ensifera ears. Each ear comprises three functional parts: two equally sized tympanal membranes, an underlying system of modified tracheal chambers, and the auditory sensory organ, the crista acustica. This organ sits within an enclosed fluid-filled channel–previously presumed to be hemolymph. The role this channel plays in insect hearing is unknown. We discovered that the fluid within the channel is not actually hemolymph, but a medium composed principally of lipid from a new class. Three-dimensional imaging of this lipid channel revealed a previously undescribed tissue structure within the channel, which we refer to as the olivarius organ. Investigations into the function of the olivarius reveal *de novo* lipid synthesis indicating that it is producing these lipids *in situ* from acetate. The auditory role of this lipid channel was investigated using Laser Doppler vibrometry of the tympanal membrane, which shows that the displacement of the membrane is significantly increased when the lipid is removed from the auditory system. Neural sensitivity of the system, however, decreased upon removal of the lipid–a surprising result considering that in a typical auditory system both the mechanical and auditory sensitivity are positively correlated. These two results coupled with 3D modelling of the auditory system lead us to hypothesize a model for weta audition, relying strongly on the presence of the lipid channel. This is the first instance of lipids being associated with an auditory system outside of the Odentocete cetaceans, demonstrating convergence for the use of lipids in hearing.

## Introduction

Hearing in insects has evolved several times independently [1]. The frequency sensitivity of these hearing systems tends to correspond to frequencies specific to predator/prey detection and intra-specific communication [1]–[3]. The most common insect ears are tympanal, comprised of three anatomically distinct functional parts – the drum-like tympanum, enlarged trachea and the tympanal receptor organs – and can occur on almost every part of the body [1], [4]. In the Ensiferan group (Insecta: Orthoptera), which includes katydids (Tettigoniidae), crickets (Gryllidae) and weta (Anostostomatidae), the ears are located on the front leg tibia [4]–[6]. Although the mechanical properties of structures within the ensiferan ear are diverse, all Ensifera with the ability to hear, do so with similar tympanal receptor organs. In Tettigoniidae [7], Anostostomatidae [8] and Haglidae [9] the tympanal organs are collectively known as the Complex Tibial Organs (CTOs), which include the subgenual organ, crista acustica, intermediate organ and accessory organ [4], [6], [10]. These organs sit within a liquid filled channel, that has been presumed to be filled with hemolymph [5], [7] – the auditory function of this channel has not been investigated. The liquid channel and organs are attached to an adapted trachea system; the level of the adaptation varies between species within the Ensidera group [7], [9], [11], [12]. In some Tettigoniidae species, these adaptations are thought to change the transmission characteristics of the incoming sound wave [7], [11], [13], [14].

The subject of this study is a species of Tree weta *Hemideina* (Ensifera: Anostostomatidae), which is endemic to New Zealand and has a fossil record dating back 270 million years. They have evolved in isolation since New Zealand was separated from Gondwanaland approximately 80 million years ago [15]. Very little is known about the hearing abilities and modes of communication of most weta species. Studies and observations indicate they communicate using both far-field hearing and vibration. Weta produce sound by stridulation [16], [17], and the calls produced are broadband with frequency peaks between 2–14 kHz [17], depending on the type of stridulation being produced. Weta are thought to produce four different behavioural stridulations [18]. However, a complete repertoire of sound is only known for one weta species, *Hemideina crassidens* [18].

*Hemideina* spp. hear over a narrow frequency range relevant to acoustic intraspecific communication [19], [20]. They hear with a typical ensiferan auditory system, comprising complex tibial organs, an adapted trachea system and associated tympanal membranes on the foreleg tibia [21]. The primary input for sound transduction is via anterior and posterior tympanal membranes [19], each of which behaves as a simple mechanical oscillator resonating with a drum-like movement [20]. The membranes are divided into two distinct regions, a pigmented thick inner plate region and a surrounding thin transparent region. The inner region oscillates as a stiff plate driven by the surrounding region creating a loading effect that may be a mechanical adaptation contributing to frequency filtering, especially at higher frequencies [20]. As the tympana vibrate in a simple uniform mode [20], the membrane itself is unlikely to contribute to any frequency discrimination.

The aim of this research was to understand the role the hemolymph channel plays in the sensitivity of an insect auditory system and if it contributes to further frequency discrimination of this auditory system. While investigating this role, we were surprised to discover that complex tibial organs are not actually bathed in hemolymph, but in a medium comprised principally of lipid from a new class as determined by mass spectrometry. Further investigations into the function of the lipid revealed a previously undescribed tissue structure, additional to the known complex tibial organs, sitting within the lipid-filled channel; we refer to this structure as the olivarius. To investigate possible associations between the olivarius and the lipid we incubated tibial sections containing intact olivarii with radiolabelled acetate, then solvent-extracted and separated the components using thin-layer chromatography followed by autoradiography. To elucidate the mechanical role of the lipid, we used Laser Doppler vibrometry to measure the mechanical response of the membrane before and after the removal of the lipid. The combination of these two results and 3D modelling of a complete auditory system leads us to hypothesize a model for weta audition.

**Figure 1:**
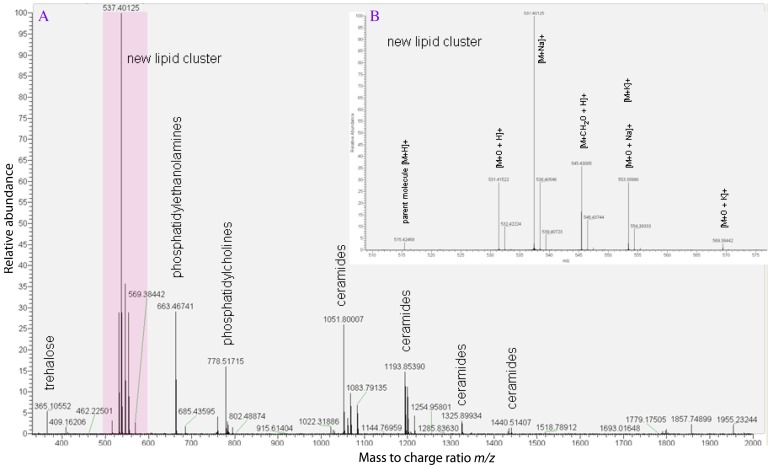
High resolution accurate mass spectrum of lipids found in the weta auditory system. (A) Full scan positive ion spectrum showing the new lipid cluster (shaded) and other more conventional lipids. (B) Insert, shows interpretation of related ions (adducts) to *m/z* 515.42468 the parent molecule.

## Results

### Lipid Composition

Surprisingly, the fluid surrounding the CTOs, is not hemolymph but lipid. Fourier transform ion cyclotron resonance mass spectrometry of the fluid within the channel shows minimal contamination from hemolymph triglycerides, possibly due to penetration of the syringe (used to remove lipid) through a thin hemolymph layer to access the lipid channel. Traces of trehalose, together with gangliosides, ceramides and phospholipids typical of membrane lipids are present together with a major cluster of cation adducts related to a protonated parent ion at *m/z* 515.42468 (Fig. 1A). Accurate tandem mass analysis of these ions revealed a lipid constituent not corresponding to any hitherto known lipid class (Fig. 1B), indicating that this lipid is likely to be a new category of lipid. A large positive mass defect value for the atomic mass indicates a high alkyl and probably nitrogen content. Fragmentation analysis of the parent ion points to sequential loss of 2× *m/z* 168.18702 (corresponding to C_12_H_24_) probably resulting from allylic cleavage of the terminal tail of two constituent unsaturated fatty acid or sphingosine units.

**Figure 2:**
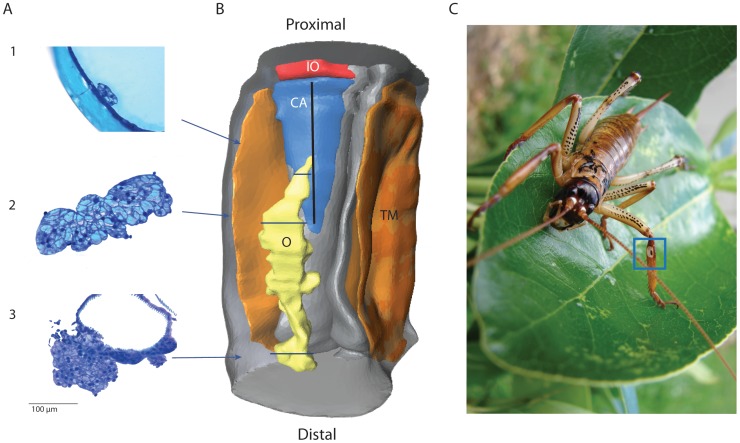
The structure and placement of the olivarius organ in relation to the known auditory organs. (A) Cross sections (5 µm) showing a proximal view (1), middle view (2) and distal view (3) of the olivarius stained with Toluidine blue. Arrows point to blue lines corresponding to location of the section on the model. Scale 100 µm. (B) A 3D reconstruction from 519 cross sections, 5 µm thick constructed in Amira, shows the placement and size of the olivarius (O) is shown in yellow in relation to known auditory structures; the crista acustica (CA in blue) and associated sensilla (not modeled, the presentation is represented by the black line running down the centre of the CA), tympanal membranes (TM in orange), trachea (grey) and intermediate organ (IO in red). The subgenual organ, which sits proximal to the intermediate organ, is not revealed. The length of the model is approximately 2.5 mm. (C) A female *Hemideina thoracica*, the blue box boarders the location of the auditory structures (the white tympanal membranes can be seen) and the area model in the 3D reconstruction.

**Figure 3:**
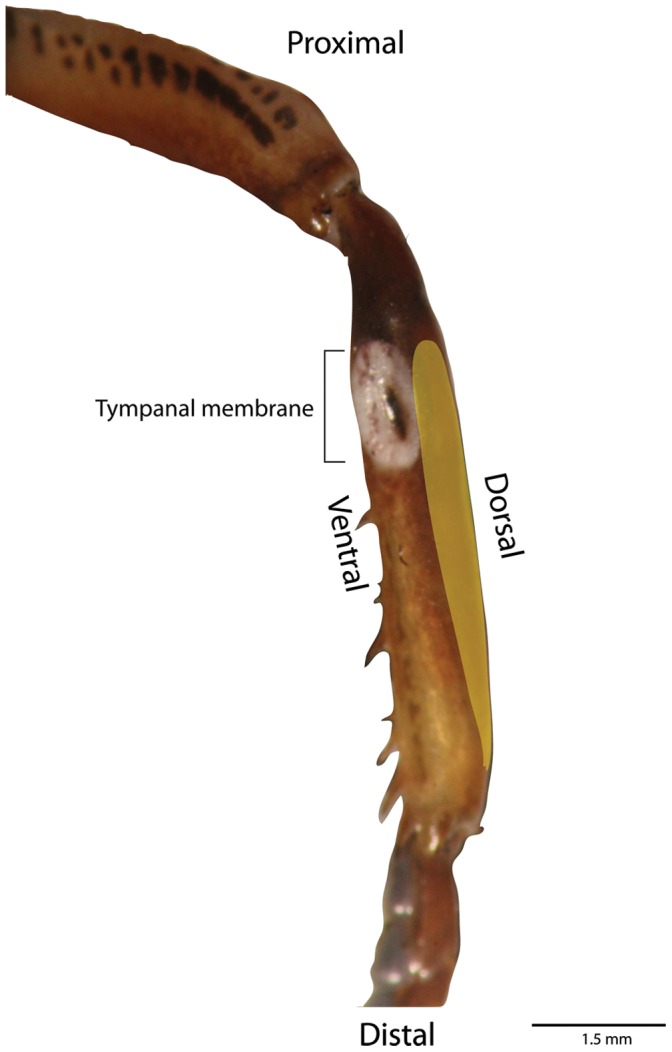
The front leg tibia showing the tympanum membrane with the lipid channel (highlighted in yellow) running from the top of the tympanum to the bottom of the tibia along the dorsal side of the leg.

### Morphology of the Olivarius and Lipid Channel

Three-dimensional reconstruction of the complete auditory system of *H. thoracica* revealed an additional structure that has not been previously described. The structure, referred to here as the olivarius (Fig. 2), lies within the same fluid channel as the crista acustica and the intermediate organ. Investigation of this channel using dissection revealed the lipid is contained in a channel-like structure (Fig. 3). The olivarius is spring-like in appearance, and attached at its proximal end to the dorsal wall of the channel, against the leg cuticle. It is attached at its distal end to the ventral side of the channel at the location of an enlarged region of trachea known as the anterior tympanal vesicle. The middle region is suspended within the lipid. The structure is delicate and of similar size to the other auditory organs, being 1.5 mm in length. Staining with toluidine blue (diluted 1∶1 with 70% ethanol) shows there are no neurons associated with the structure. Visually, the tissue appears to contain secretory cells housing lipid droplets.

**Figure 4:**
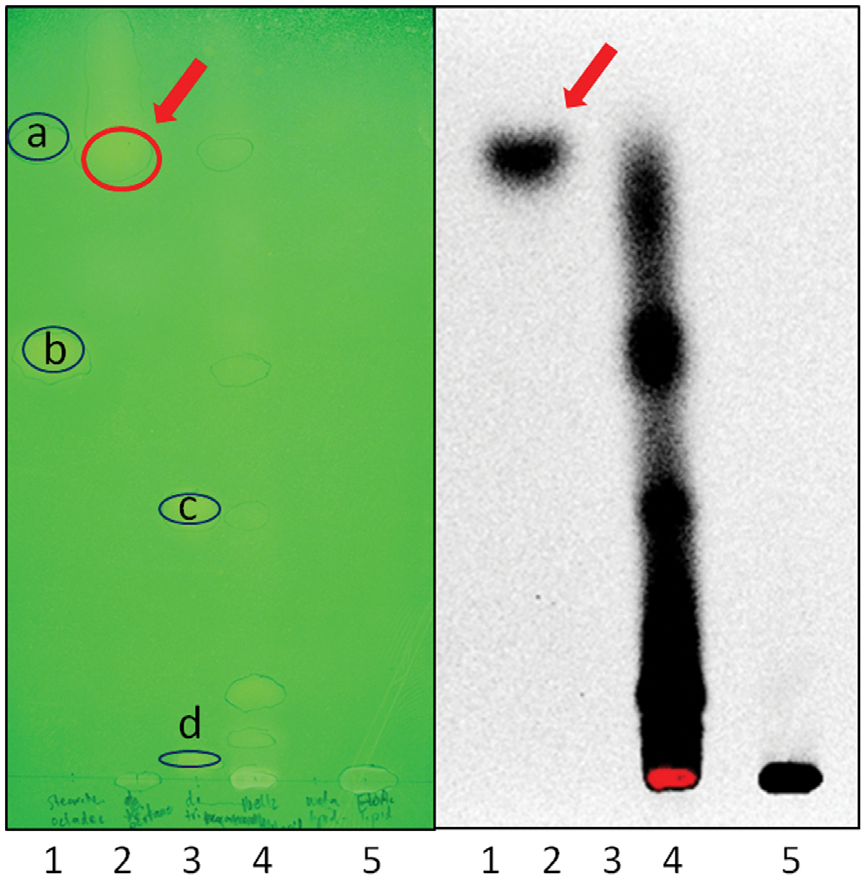
TLC plate of extracted weta leg segments containing the olivarius organ incubated with radiolabelled acetate, following extraction and solvent partitioning. The non polar phase was applied to silica solid phase extraction (SPE) and eluted fractions were applied to TLC and developed in hexane/ethyl acetate/acetic acid (20∶1:0.1 v/v/v). (A) Plate sprayed with 1% berberine sulphate in acetone/methanol (1∶1 v/v) showing standards: lane 1a, octacosane; lane 1b, tripalmitin; lane 3c, stearic acid; lane 3d, dipalmitin. Sequential SPE eluant fractions from weta leg extracts: lane 2, pentane; lane 4, dichloromethane/methanol 20∶1; lane 5, ethyl acetate. (B). The corresponding autoradiogram of the plate as a storage phosphor image after contact for 2.5 weeks. The red circle and arrows indicate the major lipid spot present as mass (A) and labeled with [^14^C]-acetate.

### Lipid Biosynthesis and Potential Function of the Olivarius

The lipid is highly hydrophobic and not water miscible, the predominant component possessing a polarity similar to long chain hydrocarbons and considerably more non polar than triglycerides, ceramides and sphingolipids (Fig. 4A). Incubation of intact olivarii in foreleg segments with radiolabelled acetate followed by solvent extraction and thin-layer chromatography with subsequent autoradiography shows this region is capable of *de novo* biosynthesis of this lipid from acetate (Fig. 4B). Since the majority of lipids in insects are synthesised in the fat body, this finding of *de novo* lipid synthesis in leg segments is significant and together with its histological appearance demonstrates that the olivarius is most likely functioning to secrete this specialised lipid into the channel. However, due to the delicate nature of the olivarius, the foreleg segment incubated also included the other complex tibial organs so their contribution cannot be totally discounted.

**Figure 5:**
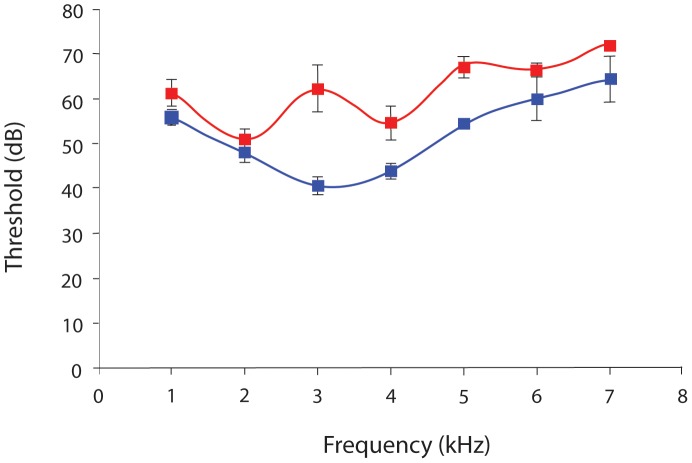
Auditory neural tuning of the tympanal organ when the lipids of the fluid channel are removed (red line). Frequency of best sensitivity is at 2 kHz, with a threshold at 55 dB SPL (*n* = 8). A threshold curve of an intact system (*n* = 9) has been overlaid to show the reduction in sensitivity (blue line). The error bars represent the standard error. Note, for some data points the error bar is too small to be seen.

### Auditory and Mechanical Sensitivity

To ascertain the role of this lipid in audition, we performed both mechanical and electrophysiological experiments. Auditory threshold experiments were performed before and after the removal of lipid from the channel surrounding the auditory organs (Fig. 5). Before the removal of the lipid the threshold curve is as expected and in line with previous published results [19], [20]. However after the removal of the lipid the structure of the threshold curve is lost resulting in the frequency of peak sensitivity decreasing from 3 kHz to 2 kHz, with a concomitant decrease in sensitivity to all frequencies (Fig. 5). The hearing threshold at the peak frequency of 3 kHz changed from 40.69±1.97 dB SPL with the lipid channel intact to 62.27±5.11 dB SPL once the lipid was removed (Fig. 5).

**Figure 6:**
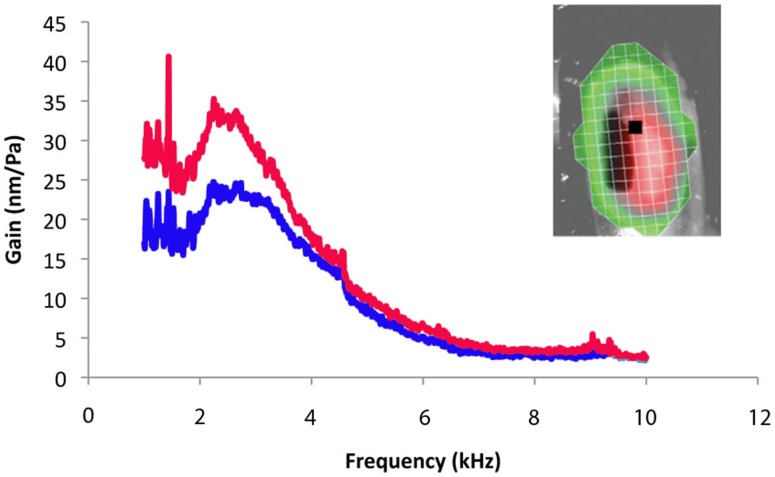
The mechanical gain (nm/Pa) of the tympana membrane at the point of maximum deflection, on the ventral side of the pigmented plate . Black Square represents point of maximum deflection (insert). Displacement at the maximum point with lipid channel intact (red line) and then the lipid removed (blue line), *n* = 17.

Surprisingly, the decrease in auditory sensitivity was not due to a decrease in mechanical tympanic vibration. On the weta tympanum, the point of maximum tympanal displacement due to sound is on the ventral side of the pigmented thickened plate (Fig. 6, insert). This area corresponds to the point where the enlarged trachea (auditory vesicles) is in contact with the tympanal membrane. The response of an intact auditory system (before lipid was removed) to broadband frequency sweeps (1–8 kHz) showed a maximum gain of 24.73 nm/Pa ±2.29 SE at 2237.5 Hz (*n* = 17). When the lipid was removed there was a 30% increase at the point of maximum displacement to 35.25 nm/Pa ±4.08 SE at 2250 Hz (*n* = 17), i.e. the tympanum moves more without lipid, even though neural sensitivity is decreased. There was no change to the peak frequency. To further highlight the relevance of the lipid, a delicate experiment demonstrated that the change in gain was reversible when lipid was returned to the channel. With the lipid channel intact displacement of the membrane at its peak frequency was 24.26 nm/Pa. With the lipid removed the gain increased to 41.78 nm/Pa. Reintroducing the lipid back into the channel was difficult but possible, and the mechanical gain subsequently reduced to a near normal level of 29.52 nm/Pa at this frequency.

**Figure 7:**
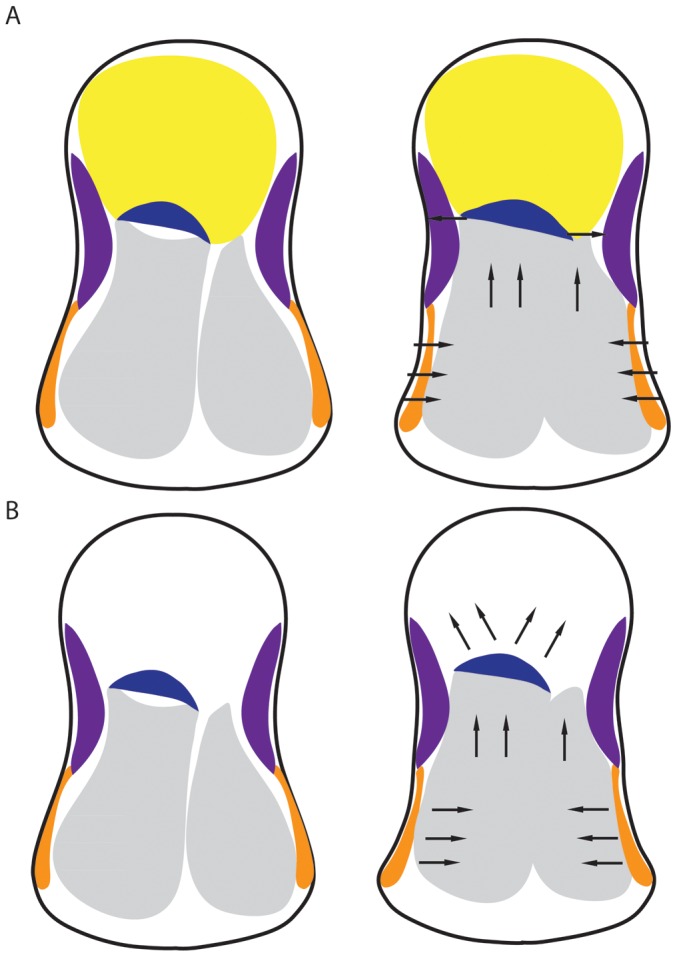
The function of the lipid channel in sound transmission. Black arrows show direction of movement. In an intact system (A) pressure displacement due to a sound wave causes the thin region of the tympanal membranes (orange) to oscillate. The two enlarged tracheal vesicles between the membranes act like bellows, displacing the air within them dorsally toward the crista acustica (dark blue), but the lipid channel (yellow) acts as a solid mass preventing the crista acustica from moving dorsally, thus this force is sideways, stretching the receptor cell on the crista acustica. The thickened inner plate of the membrane (purple) acts as a further damper by restricting the amount of sideways movement at this point. When the lipid channel has been removed (B) the crista acustica is free to move dorsally with the trachea thus reducing the sensitivity of the system, as more movement is required to stretch the receptor cells laterally.

## Discussion

Three-dimensional computer reconstructions of the weta auditory system have revealed the olivarius, a structure that sits within the same fluid channel as the other auditory organs. The channel, which stretches the length of the tibia, is filled with a lipid from a previously undescribed lipid class. The olivarius from its histological appearance is most likely responsible for producing this auditory lipid *in situ* from acetate indicating that it is able to undertake *de novo* lipid synthesis, distinct from the fat body. In insects most of the lipid biosynthesis takes place within the fat body located within the abdomen, where it can be considered functionally analogous to the liver and adipose tissue, serving as the centre for lipid biosynthesis and storage [22], [23]. In our case, the channel is isolated from the open circulatory system, so lipids especially specialized ones are unlikely to be sourced from the fat body via the hemolymph.

Although we have only described the olivarius and lipid channel within this one *Hemideina* species, a similar liquid channel has been described in bushcrickets (Tettigonidae) as being hemolymph-filled [7]. However, since there has been no analysis of this liquid to confirm that it is actually hemolymph, we anticipate that the same lipid filled channel and olivarius will be seen in other hearing Ensifera. As in *Hemideina* it is an isolated channel being enclosed distally by expanded trachea. Proximally the hemolymph channel is bordered by the subgenual organ [7].

Our study is the only example of a lipid being associated with an auditory system outside of the cetaceans, and argues a case of convergent evolution for the use of lipid as part of auditory function. However, the lipids contained in the cetacean and weta auditory systems are distinctly different. The cetacean melon and mandibular fat bodies are composed primarily of triacylglycerols, containing short- and medium-chain (C_5_–C_12_) acids made predominantly of isovaleric acid, in the form of 1,3-diisovaleroylacylglycerols [24]; a lipid rarely found in other fatty tissues [24]. The lipids in the melon serve as a lens to focus and project sound into the marine environment [24]–[26], and due to their similar density with water do not act as an impedance barrier thus making transmission highly effective [26]. The lipids of the mandibular fat bodies of cetaceans are also thought to enhance transmission of sound to the auditory receptors [27]–[30]. These cetacean fat bodies – cavities located in the posterior third of the mandible – are filled with lipids and connective tissue and this area of the dolphin jaw is most sensitive to auditory stimuli [27]. The lipids of *H. thoracica* are also unique, containing substantial alkyl regions (C_12_H_24_) from an unknown lipid class with a polarity between linear hydrocarbons and triglycerides. Results from our study indicate that the lipid plays a different role in the auditory system of the weta compared to cetaceans. Laser Doppler vibrometry measurement of the oscillating tympanic membrane shows that, when the lipid is removed from the auditory system, the displacement of the membranes is significantly increased. The auditory threshold, however, is increased upon removal of the lipid. For a typical auditory system where stimulus amplitude and auditory sensitivity are positively correlated, this result is, at first glance, counter-intuitive. Reconciling these two results leads us to postulate the following model for weta audition.

In an intact system (Fig. 7A) the thinned region of the tympanal membranes will oscillate in response to the pressure differential caused by a sound wave. Compression of the two tympana will be correlated with increased tracheal pressure. The two enlarged trachea vesicles between the membranes will act like bellows, expanding dorsally, such that the crista acustica experiences a compression force. The lipid channel would therefore act as a solid mass preventing the crista acustica (and trachea) from moving dorsally. Instead, the trachea is constrained to expand laterally, stretching out the ends of the crista acustica and thus displacing the mechano-receptors to generate action potentials. If the lipid within the channel is removed (Fig. 7B), the crista acustica is free to move dorsally with the trachea. It will therefore not undergo lateral deformation so readily. In this case, the removal of the lipid will allow the mechanical system to vibrate with a larger displacement (as seen in the vibrometry measurements), but the reduction of lateral stimulation of the crista acustica means that the electrophysiological sensitivity is reduced (as seen in the electrophysiological recordings). Udayashanker et al [31], recently showed in a species of Tettigoniidae the movement of the crista acustica in response to sound was in the form of a tonotopically ordered travelling wave covering the length of the organ. The travelling wave in conjunction with the lipid may thus affect frequency sensitivity.

Questions still remain, particularly the reason for why a lipid is used instead of, for example hemolymph, or whether the function is purely mechanical. It would be interesting to see if the material properties of this lipid are conducive to further efficiencies in this mechanical system. Comparative studies with other ensiferans could reveal further insights. Both these points constitute exciting avenues for future research. It is clear that the discovery of lipids associated with hearing in an insect auditory system offers an ideal opportunity to further explore the role of lipids in hearing systems, and further our understanding of the different acoustic capabilities relating to lipids in Nature.

## Methods and Materials

### Animals

Adult male and female *H. thoracica* were collected from native bush areas around Auckland, New Zealand. No specific permits were required for the collection and maintenance of insects and the subsequent laboratory studies. Collection was undertaken primarily on private land belonging to KFL. Other sources were from public land for which permits were not required. *H. thoracica* is not an endangered or protected species. Animals were kept in a 1 m x 1 m x 1 m enclosure and provided with a diet of New Zealand native plant material.

### 3D Reconstruction of Complete Auditory System

#### Histology sections

The tibiae from the forelegs of five animals were fixed in 4% formaldehyde for 4–6 hours. The tibia was then embedded in a block of Histocryl resin with a polymerisation time of 10 minutes. Histocryl blocks were sectioned at a 5 µm thickness on a Leica microtome. All sections were retained (resulting in 403–519 sections per ear), placed on a glass slide and stained with toluidine blue (diluted 1∶1 with 70% ethanol) and imaged on a Leica DMR fluorescence microscope equipped with a digital camera.

#### Construction of 3D model

Images of the histology sections were imported into AMIRA (v5.2, Visage Imaging Inc. San Diego CA.) stacked in order and manually aligned. Alignment of the sections was guided by a stencil grid placed on the monitor, and aided using images from an AMIRA 3D model of the foreleg built using micro-CT scanned images. Once aligned, each image was segmented into discrete components by manually tracing each structure of interest on every section. Identification of different structures was achieved using diagrams and cross sections from the sister species *Hemideina crassidens* [8] as a reference. Three-dimensional models were generated following segmentation of the different structures. The structures that were modelled were the intermediate organ (IO), crista acustica (CA), trachea, tympanal membranes and the olivarius. Two models were constructed to demonstrate repeatability; they did not differ substantially from one another.

### Lipid Composition

The contents of the lipid channel were removed from the forelegs of five weta using a fine glass capillary, after it exuded under pressure following puncture of the leg wall near the base of the tympanal membrane. The lipid channel seems to be isolated from the open circulatory system of the insect by the enlarging of the trachea and a thin membrane at the distal end, thus ensuring minimal contamination from hemolymph. The recovered exudate was then dissolved in dichloromethane/methanol (1∶1 v/v) and spotted onto a methanol-washed silica gel-coated aluminium foil sheet (Merck Kieselgel 60 F_254_) along with a range of lipids representative of several classes. These included the di- and triglycerides di- and tripalmitin, the C28 hydrocarbon octacosane, and the fatty acid stearic acid. The plate was developed in hexane/ethyl acetate/acetic acid (20∶1:0.1 v/v/v) and the lipids visualised as yellow fluorescing spots on a pale yellow background under long wavelength ultraviolet light after spraying with 1% berberine sulphate in acetone/methanol 1∶1 (v/v). Examination of a typical TLC plate showed the weta exudate running as a predominant band with an Rf between octacosane and tripalmitin (Fig. 4). The weta leg exudate is therefore highly non-polar, not water miscible and behaving as an uncharged lipid under the conditions used. Mass at lower Rf values was also seen on the berberine sprayed plate which is accounted for by the phospholipids, gangliosides and ceramides recorded by mass spectrometry.

Weta leg lipid exudate was dissolved in chloroform/methanol to give a 200-fold dilution; 10 mM ammonium acetate was added. Aliquots of this solution were infused into a ThermoFinnigan LTQ-FT (Fourier transform ion cyclotron resonance mass spectrometer) using the standard electrospray source at a flow rate of 3 µl/min. A source voltage of 4 kV and a capillary temperature of 275°C were used. High-resolution accurate mass data (<1 ppm mass error) were collected in positive and negative ion modes over the *m/z* range (300–2000), followed by an extensive collision induced dissociation analysis performed on the key ions present. A sample of the high Rf spot from TLC was recovered by elution and shown by mass spectrometry to be the specific lipid component under consideration.

### Lipid Biosynthesis and Potential Function of the Olivarius

The olivarii from three weta were incubated with radiolabelled acetate by removing a section of each foreleg, 2 mm either side of the organ, and steeping in 20 µCi [^14^C]-acetate (40 mCi/mmol) for 24 hrs in weta Ringer’s [32] solution at room temperature with gentle agitation. The medium was then extracted with hexane/ethyl acetate/methanol (5∶2:2 v/v) and centrifuged. The upper organic phase was partitioned against dilute sodium bicarbonate solution to remove remaining acetate, dried with magnesium sulphate and applied to a 1 ml silica solid phase extraction cartridge (Supelclean™, Sigma-Aldrich) and eluted sequentially with pentane, dichloromethane/methanol (20∶1 v/v) and ethyl acetate. Each fraction was concentrated under nitrogen and applied to a TLC plate and developed as above and storage phosphor autoradiography was performed using a Bio-Rad Pharos FX plus multifunction scanner.

### Threshold Curves

Male and female *H. thoracica* were mounted dorsal side up on a platform of dental wax (Surgident Periphery wax; Heraeus Kulzer, Hanau, Germany) and the mid and hind legs secured with wax. The forelegs were restricted from moving by securing the coxa and upper femur with wax. Removing a small section of cuticle located between the coxa and prothoracic ganglia exposed the leg nerve. Sound evoked responses were recorded from the N5 nerve of the foreleg with a silver hook electrode (0.005′′). Summed action potentials were amplified on AM-systems amplifier (AM-systems, Sequim, WA, USA) (high pass 300 Hz, low pass 10,000 Hz) and recorded using Spike2 data acquisition software and Micro mk11 hardware (CED Electronics, Cambridge, UK). The threshold dB SPL was defined when a recording was at least one deviation above the average number of action potentials in pre-stimulus recordings. Thresholds were confirmed using the headphone method described by Autrum [33].

Stimuli consisted of 40 ms tones (with 0.4 ms rise and fall times) ranging from 1–10 kHz in 1 kHz intervals. Tones were presented ipsilateral to the ear with decreasing intensity, and in random order. Tones were generated using a custom-written MATLAB (Mathworks, Natick MA) script and presentation through the Micro mk11 DAC output channel connected to a 4′′ Kevlar/Dome tweeter speaker (Jaycar Electronics, Rydalmere, Australia). Stimulus amplitude was measured by recording each tone at the threshold level using a ½ inch condenser microphone (Bruel & Kjaer 4939, Naerum, Denmark) placed next to the ear and calibrated with a Bruel & Kjaer acoustical calibrator (type 421; 94 dB SPL at 1000 Hz).

Threshold curves were generated with the auditory system intact, and then again with the lipid removed. The lipid removal process began by making a small hole into the cuticle covering the centre of the lipid channel. A small hollow glass electrode, held at the entrance of the hole, was then used to remove lipid by capillary action. The hole was then resealed with wax.

### Mechanical Measurements

Twenty *H. thoracica* were transported to the University of Strathclyde, UK, where laser Doppler vibrometry experiments were carried out. Once in the UK, animals were placed in plastic containers and provided with a diet of apple, carrot, lettuce and fish food flakes.

For laser Doppler vibrometry scanning, animals were encased in Blu-Tack® (Bostik-Findlay, Stafford, UK) to restrict movement and only the foreleg to be scanned was kept free. Animals were then mounted on a metal platform with the free leg secured to the side of the platform. The sound-induced mechanical response of the tympanum of an intact system, before and after removal of lipid, was measured at the point of maximum deflection, located on the ventral side of the inner plate. Lipid was removed from the lipid channel using a 1 ml syringe with a 30 gauge needle inserted into the lipid channel 1–2 mm below the tympanal membrane. As a control the lipid was held in the syringe and then injected back into the lipid channel. To prevent lipid leaking back out of the aperture the injection hole was sealed with superglue (RS Cyanoacrylate adhesive SMT, wire tack kit RS-473398, RS Superglue Activator RS-473439, UK).

Vibrational responses to sound were measured using a microscanning laser Doppler vibrometer (LDV, Polytec PSV-300-F, Waldbronn, Germany) with an OFV-056 scanning head fitted with a close-up attachment. Acoustic signals were generated from the PSV software-controlled digital-to-analog converter, amplified (Sony, TAFE570: Tokyo, Japan) and presented through a loudspeaker (Heil AMT, ESS Laboratories, South El Monte, CA, USA) positioned *ca* 300 mm from the tympanum being measured. Amplitude was measured using a 1/8′′ condenser microphone and preamplifier (Brüel & Kæjr: 4138 microphone, Nexus 2690 preamplifier: Nærum, Denmark) positioned 1–5 mm from the tympanum. Microphone and vibrometry signals were simultaneously recorded by the LDV system. Acoustic signals used were periodic chirps - linear frequency-modulated sweeps. The spectrum of the stimulus was corrected to be flat at 20 mPa (60 dB SPL) over 1–10 kHz. The amount of unrelated noise was estimated by calculating the magnitude-squared coherence. Data were considered of sufficient quality when coherence exceeded 85%.

Analytical and response signals were processed, analysed, stored and displayed using the LDV software (PSV v8.8). For each signal, a frequency spectrum was generated using a fast Fourier transform (FFT) with a rectangular window and a resolution of 12.5 Hz. From this, the transfer function of the membrane displacement to SPL (Pa) was calculated to produce the amplitude gain and phase response of the system at different frequencies.
